# A monomeric StayGold fluorescent protein

**DOI:** 10.1038/s41587-023-02018-w

**Published:** 2023-12-11

**Authors:** Esther Ivorra-Molla, Dipayan Akhuli, Martin B. L. McAndrew, William Scott, Lokesh Kumar, Saravanan Palani, Masanori Mishima, Allister Crow, Mohan K. Balasubramanian

**Affiliations:** 1 Centre for Mechanochemical Cell Biology and Division of Biomedical Sciences, Warwick Medical School, University of Warwick, Coventry, UK; 2 Department of Biochemistry, Indian Institute of Science, Bangalore, India; 3 School of Life Sciences, University of Warwick, Coventry, UK; 4 Warwick Medical School, University of Warwick, Coventry, UK

**Keywords:** X-ray crystallography, Fluorescence imaging

## Abstract

StayGold is an exceptionally bright and stable fluorescent protein that is highly resistant to photobleaching. Despite favorable fluorescence properties, use of StayGold as a fluorescent tag is limited because it forms a natural dimer. Here we report the 1.6 Å structure of StayGold and generate a derivative, mStayGold, that retains the brightness and photostability of the original protein while being fully monomeric.

## Abstract (web summary)

The bright, photostable fluorescent protein StayGold is converted into a monomer.

## Main

Fluorescent proteins, starting from the *Aequorea victoria* green fluorescent protein (GFP) to its variants and other fluorescent proteins, have advanced the study of biological processes across scales, from single molecules to whole-tissue behavior. The recently developed fluorescent protein StayGold (an engineered variant of a GFP from *Cytaeis uchidae*) is of special interest because of its brightness and exceptional resistance to photobleaching, with potential uses from single particle to volumetric imaging^1^.

**Fig. 1:**
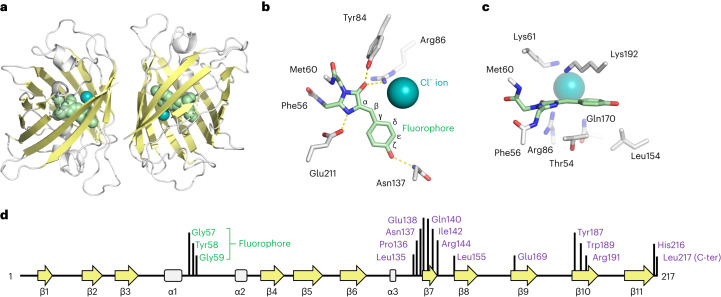
Structure of the StayGold fluorescent protein at 1.6 Å resolution. **a**, Overall structure of the StayGold dimer. **b**, Top–down view of the StayGold fluorophore and chloride ion. **c**, Side-on view of the StayGold fluorophore highlighting residues above and below the plane of the fluorophore. **d**, Secondary structure arrangement in the StayGold peptide. Locations of residues forming the fluorophore are shown in green and residues forming the dimer interface are purple.

We determined the structure of StayGold using X-ray crystallography. Crystals of StayGold belong to space group P6_1_ and diffract to 1.6 Å resolution. The structure of the StayGold dimer is shown in Fig. 1a with data collection and refinement statistics in Supplementary Table 1. The underpinning electron density is of high quality throughout (Supplementary Fig. 1). Each StayGold monomer is composed of an 11-stranded β-barrel that is almost identical to the well-characterized GFP of *A. victoria*^2^. The StayGold fluorophore is located at the center of the barrel and is formed from residues Gly57, Tyr58 and Gly59 (Fig. 1b). A notable feature of StayGold is the presence of a chloride ion immediately beside the fluorophore. The chloride ion is located within the same plane as the fluorophore and is held in place by electrostatic interactions with Lys61 and Lys192 on one side and Arg86 on the other. The chloride ion interacts with the carbonyl oxygen of the fluorophore and is close to the hydrogens on both the C_β_ and C_δ_ atoms of what was originally Tyr58. An equivalent chloride ion is also found in mNeonGreen^3^. Further experiments will be needed to understand the role of chloride in the brightness of StayGold.

Several residues on the inside of the StayGold protein barrel make hydrogen bonds with the fluorophore. These residues are Tyr84, His102, Asn137 and Glu211. The sidechains of Thr54, Val139, Leu154, Val152 and Lys192 are also positioned close to the fluorophore and could perhaps be targeted by mutagenesis campaigns seeking to generate new StayGold variants.

A current limitation of StayGold is that it forms a natural homodimer. Many researchers are wary of using StayGold as a fluorescent fusion protein because unintended dimerization of StayGold-tagged molecules might lead to experimental artifacts. Dimerization-induced artifacts are well documented in the fluorescent protein literature^4,5^. One solution to dimerization is to use tandem pairs of StayGold monomers in protein fusions^1^. However, tandem fusions do not necessarily preclude intermolecular dimerization, and the size of the tandem StayGold tag (~50 kDa) is prohibitively large. Furthermore, community investment in the future development of StayGold proteins for use in applications such as protein–protein Förster/fluorescence resonance energy transfer (FRET), multicolor imaging, split-fluorescent proteins or as biosensors (all mature technologies in conventional fluorescent protein systems) would be greatly accelerated if a monomeric form of StayGold were available. We therefore used our crystal structure to engineer a monomeric form of StayGold.

**Fig. 2:**
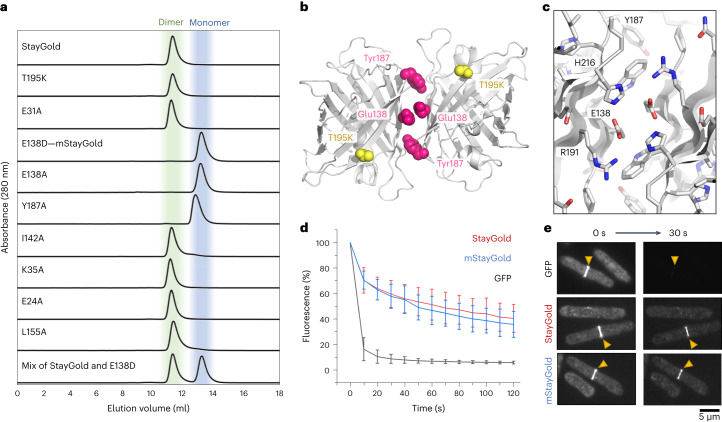
Rational design of a monomeric StayGold derivative for use in biological research. **a**, Size-exclusion chromatography showing the oligomeric state of StayGold and its variants. A mixture of StayGold and mStayGold (that is, E138D) is used to demonstrate their separability. **b**, Location of two monomerizing mutations (E138D and Y187A) and nonmonomerizing (T195K) mutation in the context of the StayGold dimer. **c**, Close-up view of the StayGold dimer interface. **d**, Photostability of StayGold and mStayGold relative to GFP. Fluorescence is given as a percentage of the initial brightness of the *S. pombe* cytokinetic ring for cells expressing the indicated fluorescent protein fusion to the myosin light chain. Error bars indicate s.d. (*n* = 20). **e**, Representative images showing photobleaching for *S. pombe* cytokinetic ring using GFP, StayGold or mStayGold protein fusions. Yellow triangles indicate the cytokinetic ring.

Using the computer program PISA^6^, we initially identified two interfaces in the crystal structure that could conceivably represent the dimer interface. Neither interface is the same as the dimer interface found in the original GFP, and both are predicted by PISA to be only marginally stable. To experimentally distinguish the biologically relevant dimeric interface of StayGold from crystal contacts, we performed size-exclusion chromatography (SEC) experiments using StayGold variants with engineered single amino acid substitutions located at each of the potential interfaces (Fig. 2a and Supplementary Table 2). As expected from the crystal structure, the T195K variant (equivalent to A206K in *A. victoria* GFP^4^) does not monomerize the StayGold protein, confirming that the StayGold dimer is distinct. We also ruled out a potential interface formed by residues E24, E31 and K35 because alanine substitutions at these sites did not affect the protein’s oligomeric state. Conversely, mutations in the remaining interface did disrupt oligomerization; both I142A and L155A have partly monomeric fractions, while E138A, E138D and Y187A variants are truly monomeric (Fig. 2b).

Integrating the peak areas in our size-exclusion chromatography data shows that the E138D mutant is more than 99% monomer, while the original StayGold is ~98% dimer (see Supplementary Table 2 for an analysis of all 11 variants). Size-exclusion measurements at higher protein concentrations show no evidence of concentration-dependent dimerization for the E138D and Y187A variants at 150 μM (Supplementary Fig. 2). Comparison with protein molecular weight standards (including superfolder GFP (sfGFP) variants and eGFP (enhanced GFP)) suggests an apparent molecular weight of 20 kDa for mStayGold and 40 kDa for StayGold consistent with monomer (26 kDa) and dimer (52 kDa), respectively. Our mutagenesis experiments therefore confirm the arrangement depicted in Fig. 2a as the functionally relevant dimer in StayGold. We note that similar interfaces have been observed for KillerRed^7^ (a dimer) and the red fluorescent protein of Discosoma DsRed^8^ (a tetramer) among others.

The monomerizing effect of the E138D mutation can be understood from the StayGold structure; shortening of the negatively charged side chain using a Glu-to-Asp substitution forces the carboxyl groups at the tip of each side chain into proximity causing repulsion between monomers (Fig. 2c). Mutation of Glu138 also disrupts stabilizing hydrogen bonding interactions with His216 and Arg191 that are colocated at the dimer interface (Fig. 2c). Indeed, Arg191 forms an important interprotein salt-bridge with the StayGold C-terminus—which may explain why both E138D and E138A mutations are effective in disrupting the dimer.

Having established a monomeric form of StayGold, we next compared its fluorescence properties to the original dimer (Supplementary Figs. 3–7 and Supplementary Table 3). We find that StayGold and mStayGold have near-identical excitation (Ex.) and emission (Em.) maxima (Ex., 497 nm and Em., 504 nm) and similar extinction coefficients (165,000 M^−1^ cm^−1^ for StayGold and 145,000 M^−1^ cm^−1^ for E138D mStayGold). Quantum yields for StayGold and mStayGold are similar to one another (0.91 and 0.87) and high in comparison to sfGFP (0.63). Both proteins are stable over a broad pH range with an apparent p*K*_*a*_ of 4.0 (StayGold) and 4.6 (mStayGold) likely corresponding to protonation of the fluorophore. The half pH unit difference in p*K*_*a*_ was the same for both E138D and Y187A variants suggesting the shift is due to the impact of monomerization itself rather than the nature of the amino acid substitutions used to produce monomers. No difference in the rates of chromophore maturation was observed between StayGold and mStayGold (Supplementary Fig. 8). We therefore find that the monomerizing mutations presented here do not adversely affect the fluorescence properties of mStayGold.

We then assessed the photostability of mStayGold in live and fixed cells. We first fused StayGold, mStayGold or a monomeric GFP to the myosin light chain in *Schizosaccharomyces pombe* and imaged the cytokinetic actinomyosin ring using live cell spinning disc confocal microscopy (Supplementary Video). At full laser intensity, the GFP fusions were quickly photobleached, losing ~90% of their initial emission intensity after 30 s. By contrast, both StayGold and mStayGold lost only ~40% brightness in the same period (Fig. 2d,e; further repeats shown in Supplementary Fig. 9). In a second imaging experiment, we observed the cytoskeleton of fixed human retinal pigment epithelial-1 (RPE-1) cells expressing the actin-binding protein tropomyosin^9^ (a naturally dimeric coiled-coil protein^10^) fused to either mNeonGreen^11^, StayGold^1^ or mStayGold (Supplementary Fig. 10). The use of fixed cells confirmed that the long-lived brightness of StayGold and mStayGold was intrinsic to these proteins and not due to newly folded fluorescent molecules replenishing those lost to photobleaching. Fixed cells also eliminate the issue of protein degradation affecting bleaching measurements. Finally, we monitored photobleaching for freely expressed StayGold and mStayGold in immortalized human retinal cells (RPE-1) and human embryonic kidney (HEK293T) cells (Supplementary Fig. 11a,b). StayGold and mStayGold maintained their fluorescence substantially longer than mNeonGreen. No difference in cellular brightness was observed between StayGold and the monomeric E138D variant, although their brightness was lower than mNeonGreen (Supplementary Fig. 11c). The apparent cellular brightness can be affected by various factors such as rates of transcription, translation, folding, chromophore maturation and protein degradation. Further adaptation might benefit the use of StayGold in applications where the highest expression level of the free form is preferable—such as a cell-fate tracer.

To probe photostability in vitro, we made photobleaching measurements using purified fluorescent proteins immobilized in polyacrylamide (Supplementary Fig. 12). These experiments exclude the effects of differential protein expression (or turnover) allowing us to focus on the photostability of the proteins themselves. Consistent with the data obtained in cells, we find that monomeric StayGold variants last substantially longer under laser illumination than conventional fluorescent proteins such as sfGFP. For example, we measured a half-life of ~70 s for mStayGold E138D compared to 11 s for sfGFP (Supplementary Fig. 12a). Superior photostability of StayGold and mStayGold was also observed under widefield illumination with a metal halide source (Supplementary Fig. 12b).

While testing StayGold for an imaging application in budding yeast, we identified fusions of the original StayGold to the septin collar protein (Shs1) that resulted in frequent mis-localization events and visible aggregation (Supplementary Fig. 13). Hypothesizing that the Shs1–StayGold aggregates were caused by promiscuous dimerization of the StayGold tag, we introduced the E138D mutation. The resultant mStayGold-Shs1 fusions behave as expected, labeling the collar of budding yeast without excess aggregates. These data lend further in vivo support for the monomerizing function of the E138D mutation in StayGold. Introduction of the E138D mutation may be a useful strategy to improve existing fusion protein constructs made by early adopters of StayGold.

Finally, to establish monomerization in vivo, we performed the organized smooth endoplasmic reticulum (OSER) assay^12^ for StayGold and mStayGold E138D alongside dTomato (an established dimer) and mTurquoise (a known monomer; Supplementary Fig. 14). The data show that mStayGold E138D is monomeric, with an OSER score comparable to that of the mTurquoise control, while the original StayGold is a weak dimer, with an OSER score that is lower than mStayGold but higher than dTomato. The apparent fragility of the StayGold dimer in the OSER assay is consistent with our observation that the interface can easily be broken with single amino acid substitutions.

In conclusion, we have solved the crystal structure of StayGold and identified E138D as a mutation that renders the protein monomeric without loss of brightness or photostability. We name the E138D variant mStayGold. We anticipate that both the monomerizing mutations found in mStayGold, and the StayGold crystal structure itself will be useful tools for future development of bright fluorescent tags, FRET pairs, split-fluorescent proteins and biosensors.

Note: During the revision of our manuscript, two preprints describing distinct monomeric forms of StayGold were submitted to Research Square^13,14^.

## Methods

### Cloning, expression and purification of fluorescent proteins

StayGold and mStayGold were each cloned into pET-MCN vectors and transformed into BL21(DE3) *Escherichia coli* strain. In total, 2 l cultures at OD ~0.6 were induced overnight at 18 °C with 0.5 mM IPTG (isopropyl β-D-1-thiogalactopyranoside). Samples were purified with Ni-NTA (nickel-nitrilotriacetic acid) beads in wash buffer containing 50 mM phosphate buffer, 500 mM NaCl and 30 mM imidazole at pH 7.6. Proteins were eluted with 50 mM phosphate buffer, 500 mM NaCl and 500 mM imidazole at pH 7.6. Fluorescent proteins were then exchanged into a 20 mM HEPES (4-(2-hydroxyethyl)-1-piperazineethanesulfonic acid) pH 7.2 and 150 mM NaCl using a PD10 desalting column. sfGFP and its variants were produced similarly with mutations confirmed by sequencing.

### Size-exclusion chromatography

Size-exclusion chromatography was performed using an ÄKTA Pure FPLC with a Superdex 75 Increase 10/300 GL column. Proteins were concentrated to ~2 mg ml^−1^ (76 μM) and loaded onto the column via a 100 μl loop. The flow rate was 0.8 ml min^−1^, and the buffer used was 150 mM NaCl and 20 mM HEPES (pH 7.2). Chromatograms were baseline-corrected using the mean absorbance value measured in the first 5 ml following injection. Monomer and dimer percentages were calculated after integrating the peaks corresponding to the monomer and dimer fractions. A higher concentration run using StayGold and mStayGold was performed using 4 mg ml^−1^ (154 μM) protein. Additional runs using a mixture of bovine serum albumin (66 kDa) and hen egg white lysozyme (14 kDa), sGFP (27 kDa) or BIORAD molecular weight standards (thyroglobulin, 670 kDa; ɣ-globulin, 158 kDa; ovalbumin, 44 kDa; myoglobin, 17 kDa and vitamin B_12_, 1.35 kDa) were used for estimation of apparent molecular weights.

### Crystallization

StayGold was concentrated to 12 mg ml^−1^ in a buffer composed of 150 mM NaCl and 20 mM HEPES (pH 7.5) before setting up crystallization screens in MRC 2-drop plates using a Formulatrix NT8 robot. Crystallization used the sitting drop vapor diffusion method with 1 μl drops composed of either 2:1 or 1:2 ratios of protein solution and crystallization reagent. Crystals of StayGold grew in 1–2 d with many similar hits across the screens. The crystals used for structure determination were obtained using the SG1 HT96 Eco Screen (Molecular Dimensions) condition H4 (0.2 M sodium acetate, 25% PEG (Polyethylene glycol) 3350 and 0.1 M Bis–Tris (pH 6.5)). Crystals formed as long needles that could be broken apart for single crystal data collection.

### Structure determination using x-ray crystallography

Drops containing StayGold crystals were supplemented with a solution of 70% crystallization reagent (taken from the reservoir of the plate) and 30% glycerol, collected in litholoops and flash-frozen in liquid nitrogen. X-ray diffraction experiments were conducted at the Diamond Synchrotron using remote collection at beamline I04. Diffraction images were integrated using Dials^15^ via Diamond’s auto-processing pipeline. Subsequent processing was performed using tools from the CCP4 suite^16^. Space group determination and scaling were conducted with Aimless^17^. Phases were calculated by molecular replacement using a monomer from 5WJ2 (ref. ^18^) as the search probe. The molecular replacement solution (containing two molecules per asymmetric unit) was found using Phaser^19^ software. After an initial round of refinement in Refmac^20^, a new map was calculated and density modification (including non-crystallographic symmetry averaging, solvent flattening and histogram matching) was performed with Parrot^21^. A new model was then built into the density-modified map using Buccaneer^22^, and the model was completed using iterative rounds of model building with Coot^23^ and refinement using Refmac^20^. As refinement neared completion, water was added and the model was completed by modeling in the fluorophore and chloride ion. Later rounds of model building and refinement integrated validation tools including COOT^23^, Procheck^24^ and Rampage^25^. Protein–protein contacts were analyzed using PISA^6^ and molecular images generated using PyMOL^26^. The StayGold structure has been deposited in the protein databank^27^ with accession code 8BXT. X-ray data and refinement statistics are given in Supplementary Table 1.

### Fluorescence excitation and emission spectra

Fluorescence spectra were obtained using a Cary Eclipse fluorescence spectrophotometer using 5 nm excitation and emission slits. Datapoints were collected at 1 nm intervals using a scan rate of 600 nm min^−1^.

### Microscopy and in vivo photostability

*S. pombe* strains were generated by chromosomal integration of the plasmids pDUAL-p^adh11^-rlc1-GFP (pLK43), pDUAL-p^adh11^-rlc1-40aa-StayGold (pLK114) and pDUAL-p^adh11^-rlc1-40aa-StayGold (E138D, pLK126) at leu1-32 locus in MBY102. The plasmid, pLK43 was generated by Gibson cloning *rlc1* and fluorescent protein fragments into the pDUAL plasmid^28^. The plasmid pLK114 was generated by swapping GFP with a 40 amino acid linker (LEGSGQGPGSGQGSGSPGSGQGSGPGQGSGPGQGSGPGQG) and StayGold fragment with the help of NEBuilder HiFi DNA Assembly Master Mix (NEB, E2621L). The *rlc1*-40aa-StayGold(E138) variant was generated using site-directed mutagenesis. The cells were grown at 30 °C in YES medium.

The mammalian cell line, RPE-1 cells were transfected with one of the following six expression plasmids: (1) mNeonGreen-Tropomyosin2 (*pCMV*-mNeonGreen-40aaLinker-*TPM2.2*)^9^, (2) Tropomyosin2 StayGold dimer (*pCMV*-StayGold-40aaLinker-*TPM2.2*), (3) Tropomyosin2 StayGold monomer (*pCMV-*StayGold (E138D)-40aaLinker-*TPM2.2*) or (4–6) the equivalent versions lacking the tropomyosin sequences (which would express the unfused fluorescent proteins). The Tropomyosin2 StayGold plasmids were constructed by swapping mNeonGreen for StayGold using the NEBuilder HiFi DNA Assembly Master Mix (NEB, E2621L). The Tropomyosin2-mStayGold variant was generated by incorporating the E138D mutation using site-directed mutagenesis.

Immortalized (hTERT, human telomerase reverse transcriptase) diploid human RPE-1 cells (ATCC, CRL-4000) were cultured in Dulbecco’s modified Eagle’s medium/Nutrient Mixture F-12 Ham with 15 mM HEPES and sodium bicarbonate (Sigma-Aldrich, D6421) supplemented with 6 mM l-glutamine (Gibco, 25030-081), 10% FBS (Sigma-Aldrich, F7524), 100 U ml^−1^ penicillin and 100 µg ml^−1^ streptomycin (Gibco, 15140-122) at 37 °C under 5% CO_2_.

For transfection, RPE-1 cells were grown on ibiTreat 2 Well µ-Sildes (Ibidi, 80286) to 50% confluency. Cells were then transfected using Lipofectamine 2000 (Invitrogen, 11668-019) and Opti-MEM reduced-serum media (Gibco, 31985-062) according to the manufacturer’s instructions. Transfected RPE-1 cells were fixed in 4% paraformaldehyde/PBS and sealed with Vectashield (Vector, H-1000) 20 h post-transfection for imaging.

For measurement of the practical brightness of free StayGold, mStayGold and mNeonGreen expressed in mammalian cells, the RPE-1 cells cultured on a µ-Slide 8 Well high (Ibidi, 80806) were imaged 30 h after transfection using a DeltaVision microscope system (Applied Precision) equipped with a CoolSNAP HQ2 camera (Photometrics). The images with the GFP filter set and 32% illumination were acquired by softWoRx software (v5.5.1) using a ×10 UPlanSApo lens (Olympus; numerical aperture (NA) 0.4, air) with 0.25 s exposure in a z-stack of 10 images with 1.5 µm spacing. Fluorescent cells were detected by a custom ImageJ/Fiji script using trainable Weka segmentation^29^. The mean fluorescence intensities of each cell above the background level were statistically analyzed by R version 4.3.1 (https://www.r-project.org/).

For comparison of photostability, images were acquired with a spinning disk confocal microscope (Andor Revolution XD imaging system, equipped with ×100 oil immersion 1.45 NA Nikon Plan Apo Lambda, and a confocal unit Yokogawa CSU-X1, Andor iXon Ultra 888 EMCCD camera and Andor IQ acquisition software). The cells were imaged with a 488 nm laser at 100% power (~7.4 W cm^−2^) at 0.5-s intervals. Fluorescence intensities were measured for the Rlc1 GFP, Rlc1 StayGold dimer and Rlc1 StayGold monomer at the cytokinetic ring (for *S. pombe*) and the entire cell (for RPE-1 cells) for each slice using the image processing software Fiji (https://imagej.net/software/fiji/). The datapoints were normalized to the first intensity measurement.

### In vitro photostability

Photostability of pure proteins was measured using the method described in ref. ^5^. Photobleaching was quantified by embedding the fluorescent proteins into a polyacrylamide gel, which was polymerized between a 1.5 coverslip (Menzel, 12312128) and a glass slide. Other 1.5 coverslips were used as spacers. The final composition of the gel was 20% acrylamide (mono:bis 29:1), 150 mM NaCl and 20 mM Tris (pH 7.6). For laser illumination, the samples were observed using a CellR TIRFM system (Olympus) equipped with an iXon+ DU897 electron-multiplying CCD camera (Andor). The samples were illuminated with a 488 nm laser beam at a 0° angle of incidence. The power of light detected at a ×100 objective lens outlet was 2 mW, corresponding to illumination at 29 W cm^−^^2^ within a circular area with a radius of 46.5 µm. Similar experiments were performed under widefield illumination using a metal halide source.

### Extinction coefficient determination

Absorbance was measured with a Cary 50 Conc UV Visible Spectrophotometer. Molar extinction coefficients (*ε*) were determined by standardizing the absorbance at the chromophore absorption maxima (488 nm for sfGFP or 496 nm for StayGold and its derivatives) either with the protein concentration^5^ or with the chromophore concentration^1,30^. For the protein concentration method, the theoretical molar extinction coefficient at 280 nm (*ε*_280 theory_) was calculated (https://web.expasy.org/protparam/) based on the protein sequence (minus one tyrosine). The molar extinction coefficient per protein (*ε*_protein_) was calculated by multiplying *ε*_280 theory_ with the ratio of the measured absorbance values at 280 nm (*A*_280_) and the fluorophore absorption maxima (488 nm or 496 nm, *A*_peak_).

$${\varepsilon }_{{\rm{protein}}}={\varepsilon }_{280{\rm{theory}}}\times {A}_{{\rm{peak}}}/{A}_{280}$$

The *A*_peak_/*A*_280_ ratio was determined as the gradient of a *A*_peak_ versus *A*_280_ plot.

For the denatured-chromophore method, the absorbance at 447 nm after denaturation with 0.1 M NaOH (5 min for sfGFP and 1.5 min for StayGold and its derivatives, *A*_NaOH_) was measured as well as the absorbance at the absorption maxima under native conditions (488 nm for sfGFP or 496 nm for StayGold and its derivatives, *A*_neutral_). The chromophore-based molar extinction coefficient (*ε*_chromophore_) was then determined by multiplying the ratio of these measurements to the extinction coefficient of the isolated chromophore at 447 nm (*ε*_isolated chromophore_ = 44,000 M^−1^ cm^−1^) as described previously^1^.

$${\varepsilon }_{{\rm{chromophore}}}={\varepsilon }_{{\rm{isolated}}{\rm{chromophore}}}\times {A}_{{\rm{neutral}}}/{A}_{{\rm{NaOH}}}$$

### Quantum yield measurements

The quantum yield of each fluorescent protein was determined by comparing the ratio between the absorbance at the peak excitation and the integrated fluorescence emission, with that of fluorescein in 0.1 M NaOH (ref. ^5^). We used a quantum yield of 0.79 for fluorescein^31,32^. The fluorescence/absorbance ratios were each determined using the gradient from a plot of integrated fluorescence versus absorbance with at least 4 points with an absorbance below 0.1.

### Measurement of the pH sensitivity and apparent p*K*_*a*_

Absorption measurements were made at the peak absorbance wavelength after diluting purified proteins into preprepared buffers containing either 150 mM NaCl, 50 mM NaH_2_PO_4_ and 50 mM citrate (pH 2.5–7.5) or 150 mM NaCl, 50 mM Tris, 50 mM glycine (pH 8–11). Absorption measurements were normalized to the value at pH 7, and the apparent p*K*_*a*_ was estimated from the plot of normalized absorption versus pH.

### Chromophore maturation

Oxidative maturation of the chromophore of StayGold was assessed by preparing the cell lysate containing StayGold expressed under a hypoxic condition and monitoring the increase of the absorbance at 496 nm by the matured chromophore under an aerobic condition^18,33^. The BL21(DE3) cells transformed with a pET-based vector encoding StayGold or a nonfluorescent protein as a control were cultured in 25 ml LB (Lysogeny Broth) with kanamycin to the log phase (A600 ~0.5) and induced for the recombinant protein expression with 0.2 mM IPTG for 4 h at 22 °C with good aeration (this improves expression under hypoxia in the following step). The cells were collected by centrifugation and resuspended with 12 ml of degassed LB with IPTG and kanamycin. The aliquots were transferred to cryotubes (~2.5 ml; Greiner). The tubes were filled to the brim, capped expelling the air and incubated at 22 °C with rotation at 200 r.p.m. for 16–30 h. Cells from a cryotube were lysed with 400 µl BugBuster Protein Extraction Reagent (Millipore) and centrifuged at 20,817*g* for 5 min. The 200 µl supernatant was incubated at room temperature (24 °C) in a disposal cuvette (UVette, Eppendorf) and the absorbance spectra were acquired in a time course. The gradual increase of the turbidity was observed independently of fluorescent protein, due to the increasing scattering, which is inversely proportional to the fourth power of wavelength (the Rayleigh scattering). The fraction due to scattering in the absorbance at the peak wavelength (496 nm) was estimated by extrapolating the absorbance from 525 nm to 575 nm, where the absorbance by the chromophore is minimum, and subtracted from the raw read at the peak. The peak absorbance after the correction for scattering, *f*(*t*), was plotted against time, *t*, and fitted with *f*(*t*) = *β*_0_ − *β*_1_ exp(−*kt*) to determine the rate of maturation, *k*, by nonlinear least squares fitting using the nls() function in R version 4.3.1 (https://www.r-project.org/).

### OSER assays

OSER assays were performed as per the method discussed in ref. ^12^. Hela cells were transfected with one of the following four plasmids: CytERM-dTomato (Addgene, 98834), CytERM-mTurquoise2 (Addgene, 98833), pcDNA3-CytERM-StayGold or pcDNA3-CytERM-StayGold(E138D). The two StayGold plasmids were constructed by cloning G-blocks (Integrated DNA Technologies) into pcDNA3 using NEBuilder HiFi DNA Assembly Master Mix. For imaging, 20 h post-transfection, cell media was replaced with Leibovitz’s L-15 Medium (No Phenol Red). Images were acquired using Andor IQ3 software at either 69 nm per pixel with a Nikon Apo ×60/1.40 oil immersion objective lens or at 80 nm per pixel with a Nikon Plan Fluor ×40/1.30 oil immersion objective lens. The fluorophores were excited by laser lines at a wavelength of 405 nm (mTurquoise2), 488 nm (StayGold) or 561 nm (dTomato) as appropriate. Fixed cell images were acquired at room temperature, while live cell images were acquired at 37 °C. Z-stacks were obtained for each cell, followed by Z-projection in Fiji, and manual quantification was performed to count the number of cells with and without whorls.

### Reporting summary

Further information on research design is available in the Nature Portfolio Reporting Summary linked to this article.

## Online content

Any methods, additional references, Nature Portfolio reporting summaries, source data, extended data, supplementary information, acknowledgements, peer review information; details of author contributions and competing interests; and statements of data and code availability are available at 10.1038/s41587-023-02018-w.

## Supplementary information

Supplementary Information Supplementary Tables 1–3 and Supplementary Figs. 1–14.

Reporting Summary

Supplementary Video Photostability of StayGold, mStayGold (E138D) and GFP/mNeonGreen fusion proteins during cell imaging. The top row shows yeast cells containing the fluorescent fusions to the myosin light chain. The bottom row shows fusions to tropomyosin (Tpm2) in fixed human RPE-1 cells. Cells were exposed to 100% laser power (~7.4 W cm^−2^) for 2 min for yeast and 30 min for human cells.

Supplementary Data Supporting data for Supplementary Figs. 2–14.

## Data Availability

The structure of the StayGold dimer has been deposited at the protein databank (8BXT). Source data for biochemical and spectrometric experiments (Fig. 2a and Supplementary Figs. 2–8) and numerical data from the microscopy-based photokinetic or photobiological studies (Fig. 2d and Supplementary Figs. 9–14) are available for download. Raw images (>650 GB in total) from the microscopy-based studies (Fig. 2d and Supplementary Figs. 9–14) will be shared upon request.
