# Supplementary material for: A monomeric StayGold fluorescent protein
Source: Nat Biotechnol. Author manuscript; Available in PMC 2024 Sep 21. (PMC11392804; doi:10.1038/s41587-023-02018-w)
Supplement: Tables S1-S3; Figure S1-S14 [file EMS198644-supplement-Tables_S1_S3__Figure_S1_S14.pdf]

nature biotechnology

Brief Communication https://doi.org/10.1038/s41587-023-02018-w

A monomeric StayGold fluorescent protein

In the format provided by the
authors and unedited

Supplemental Information:
A monomeric StayGold fluorescent protein

Esther Ivorra-Molla[1*], Dipayan Akhuli[1,3*], Martin B.L. McAndrew[1,2], William Scott[1], Lokesh Kumar[1], Saravanan Palani[3], Masanori Mishima[1†], Allister Crow[2†], Mohan K. Balasubramanian[1†]

Schedule of Supplemental Information

Supplemental Tables
- Table S1: X-ray data and refinement statistics
- Table S2: Summary of Monomer and Dimer fractions for StayGold variants
- Table S3: Optical properties of StayGold derivatives

Supplemental Figures
- Figure S1: Electron density from the 1.6 Å resolution StayGold structure.
  Figure S2: Size Exclusion Chromatography (SEC) for dimeric StayGold and monomeric E138D variant (mStayGold) at high concentration.
- Figure S3: Fluorescence excitation and emission properties of StayGold and monomeric StayGold (E138D).
- Figure S4: Extinction coefficient measurements for StayGold, mStayGold and sfGFP using the denatured fluorescent protein method.
- Figure S5: Extinction coefficient measurements for StayGold, mStayGold and sfGFP using the A280 nm method.
- Figure S6: Quantum yield measurements for StayGold, mStayGold and sfGFP
- Figure S7: pH measurements and pK_a determinations.
- Figure S8: Oxidative maturation of the chromophore of StayGold in *E. coli* lysate.
- Figure S9: Additional repeat experiments for photostability measurements in live yeast.
- Figure S10: Photostability of mStaygold, StayGold, and measurements for fixed human RPE-1 cells.
- Figure S11: Photostability measurements for freely expressed StayGold, mStayGold and mNeonGreen in RPE-1 and HEK293T cells.
- Figure S12: *In vitro* photostability measurements using purified fluorescent proteins immobilised in acrylamide gel.
- Figure S13: Mis-localisation of the budding yeast septin collar protein Shs1 using a StayGold fusion is corrected by introduction of the E138D monomerising mutation.
- Figure S14: OSER assay data demonstrating *in vivo* monomerization of mStayGold.

Supplemental Tables

Table S1: Data collection and refinement statistics

	**StayGold (8BXT)**
**Data collection**	
Beam line	Diamond I04
Wavelength (Å)	0.95373
**Crystal parameters**	
Space group	$P6_1$
Unit cell dimensions (Å)	133.72, 133.72, 58.53
Unit cell angles (°)	90, 90, 120
**Reflection data***	
Resolution range (Å)	66.66-1.60 (1.63-1.60)
Unique reflections	78,841 (3,870)
R_{sym}	0.271 (2.418)
R_{pim}	0.062 (0.555)
$I/\sigma(I)$	9.1 (1.6)
$CC_{½}$	0.998 (0.695)
Completeness (%)	100.0 (100.0)
Multiplicity	20.5 (20.8)
Wilson B (Å^2)	11.5
**Refinement†**	
Resolution (Å)	66.66 – 1.60
Number of reflections	74,876
$R_{overall}$	0.163
R_{free}	0.185
Rms (bond lengths) (Å)	0.010
Rms (bond angles) (°)	1.52
**Model B-factors**	
StayGold (chain A) (Å^2)	16.8
StayGold (chain B) (Å^2)	16.8
Ethylene glycols	31.8
Waters (Å^2)	28.2
**Ramachandran statistics‡**	
Favoured (%)	98.6
Allowed (%)	1.4
Outlier (%)	0

Values in parentheses indicate the highest resolution bin.

Refinement statistics are from Refmac[1].

Ramachandran statistics as reported by Rampage[2].

Table S2: Analysis of StayGold variants

Protein Variant	Dimer (%)	Monomer (%)	Comments
StayGold	98	2	Stable, dimeric (Hirano *et al.*)[3]
Interface 1			
E24A	98	2	Stable, dimeric
E31A	98	2	Stable, dimeric
K35A	98	2	Stable, dimeric
Interface2			
E138A	3	97	Stable, mostly monomeric
E138D	1	99	Stable, monomeric (mStayGold)
I142A	92	8	Stable, partially monomeric
L155A	91	9	Stable, partially monomeric
Y187A	3	97	Stable, monomeric
W189D	-	-	Unstable, reduced fluorescence
W189E	-	-	Unstable, reduced fluorescence
Other<br>T195K	98	2	Equivalent to *A. victoria* GFP monomerising mutation (A206K)[4]. Does not monomerise Staygold.

Interface 2 is found to represent the biological unit.

Table S3: Optical properties of StayGold derivatives.

Protein	Peak Excitation Wavelength (nm)	Peak Emission Wavelength (nm)	Extinction coefficient per protein (10^3 M^{-1} cm^{-1})	Extinction Coefficient per chromophore (10^3 M^{-1} cm^{-1})	Quantum Yield	pK_a	Half life (mean ± SE) (s)
StayGold	497	504	122	166	0.92	4.0	31.6 ± 1.5
mStayGold E138D	497	505	106	145	0.87	4.6	73.5 ± 3.8
StayGold Y187A	498	505	104	147	0.92	4.6	69.8 ± 3.9
sfGFP	488	513	37	50	0.63	5.8	10.9 ± 0.3

Supplemental Figures

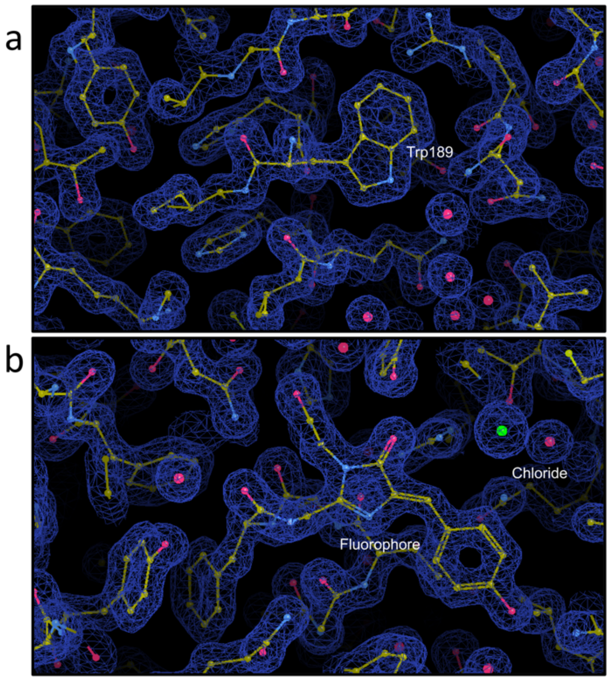

Figure S1: Electron density from the 1.6 Å resolution StayGold structure. (a) A representative view of the electron density centered on Tryptophan 189. (b) A view of the electron density for the StayGold fluorophore and chloride ion. The weighted electron density map ($2m|F_o|-d|F_c|$) is shown contoured at 1.5 σ. Red spheres indicate ordered water molecules.

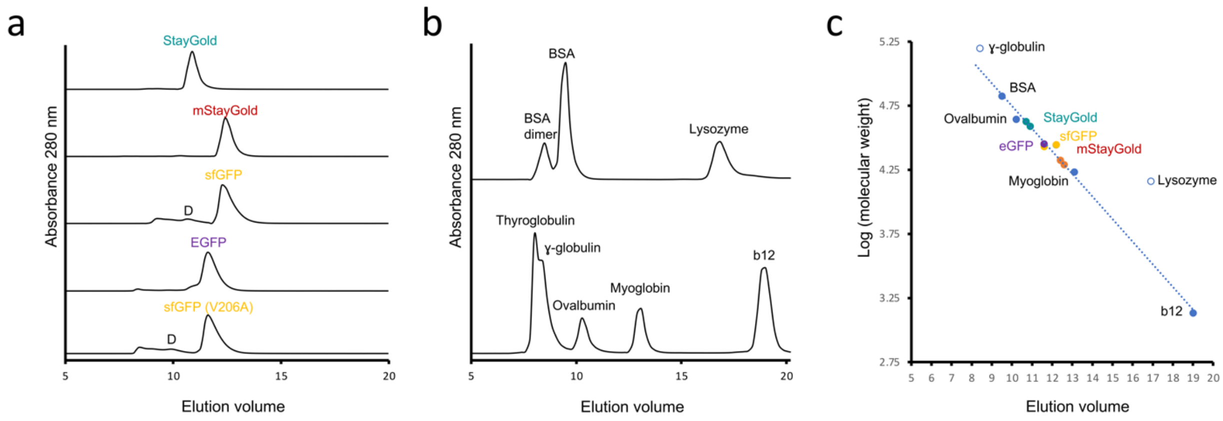

Figure S2: Size Exclusion Chromatography (SEC) for dimeric StayGold and monomeric E138D variant (mStayGold) at high concentration. (a) SEC profiles for StayGold and mStayGold at pH 7.5, 150 mM NaCl. Both samples were loaded at 4 mg/mL (154 µM). Traces for sfGFP, EGFP and a dimerization-prone sfGFP variant (V206A) are shown for comparison. A minor peak suggesting dimer for sfGFP is labelled. (b) Molecular weight standards run under the same buffer conditions. Elution volumes for Bovine Serum Albumin (66 kDa) and Hen Egg White Lysozyme (15 kDa) were 9.7 mL and 16.9 mL respectively and a BSA dimer appears in the void (~8.7 mL). Elution volumes and molecular weights for the other standards are as follows: Thyroglobulin (670 kDa) 8.2 mL, ɣ-globulin (158kDa) 8.4 mL, Ovalbumin (44 kDa) 10.2 mL, Myoglobin (17 kDa) 13.1 mL, Vitamin b_{12} (1.35 kDa) 19 mL. (c) Calibration curve fitted using molecular weight standards. Proteins larger than 70 kDa were excluded in line with column specifications. Lysozyme was excluded as an outlier). Standards are shown in blue, StayGold in teal, mStayGold in red, sfGFP in yellow and EGFP in purple. Elution volumes for sfGFP (27 kDa), EGFP (28 kDa) and sfGFP V206A (27 kDa) were 12.2 mL, 11.6 mL and 11.6 mL. Elution volumes for StayGold (10.7 mL) and mStayGold (12.6 mL) are consistent with dimer (52 kDa) and monomer (26 kDa) respectively.

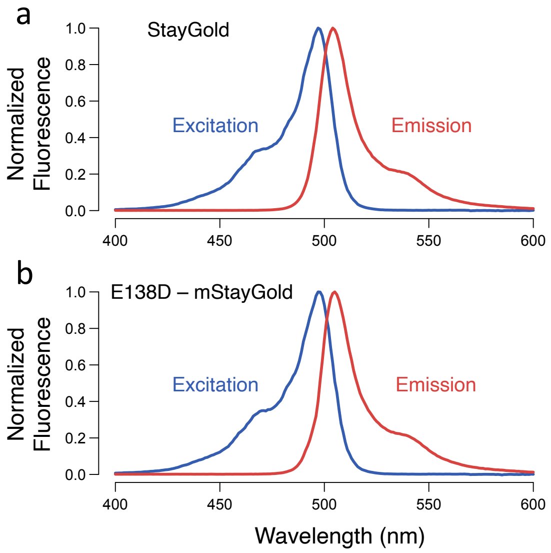

Figure S3: Fluorescence excitation and emission properties of StayGold and monomeric StayGold (E138D). For the excitation spectra, the fluorescence of StayGold (a) and monomeric StayGold (E138D) (b) was measured at 505 nm. For the emission spectra, the fluorophores were excited at 496 nm. The background signals were subtracted from the triplicate data and the mean after normalization was plotted. The excitation and emission maxima are 497 nm and 504 nm for StayGold and 497 nm and 505 nm for mStayGold (E138D).

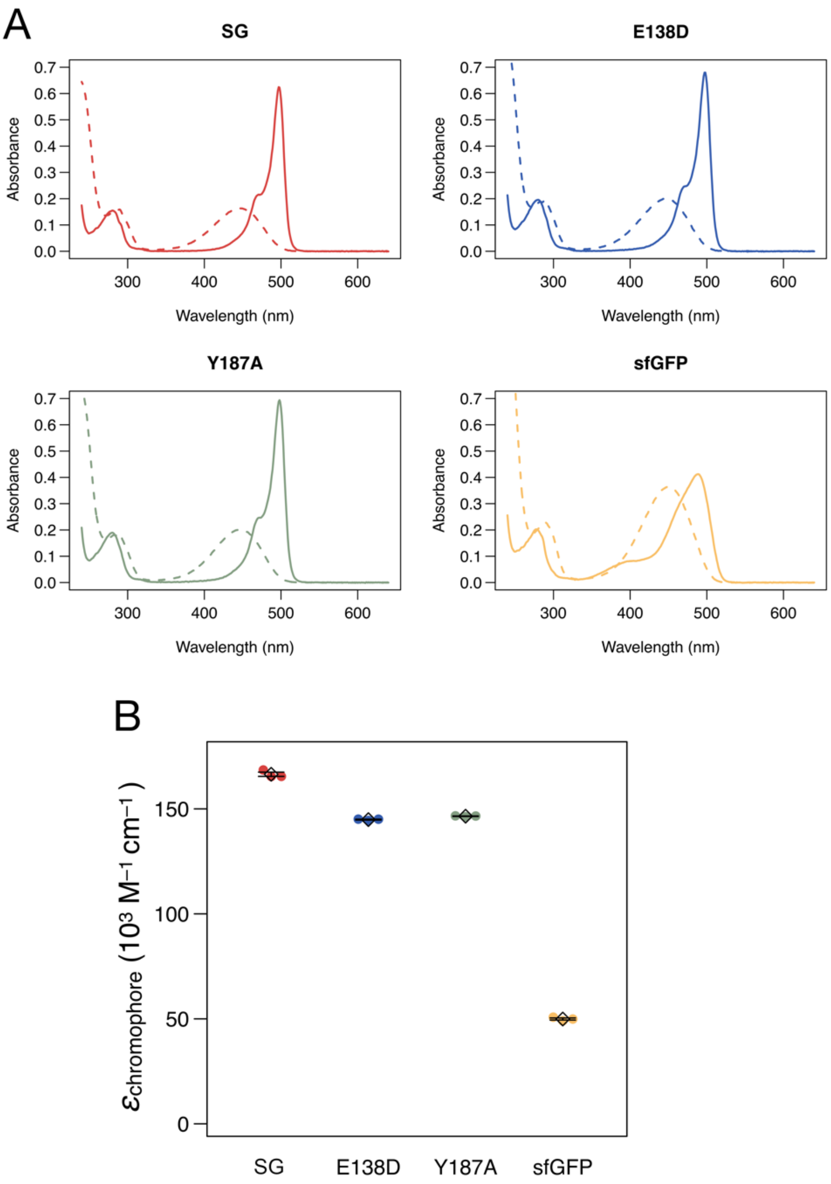

Figure S4: Extinction coefficient measurements for StayGold, mStayGold and sfGFP using the denatured fluorescent protein method. (A) Representative absorption spectra of StayGold, its derivatives, and superfolder GFP (sfGFP) measured in a neutral buffer (solid curve) and 0.1 M NaOH (dashed curve). (B) Molar extinction coefficients per chromophore at 496 nm (StayGold) or 488 nm (sfGFP). Results from three independent measurements are displayed with their mean and standard error.

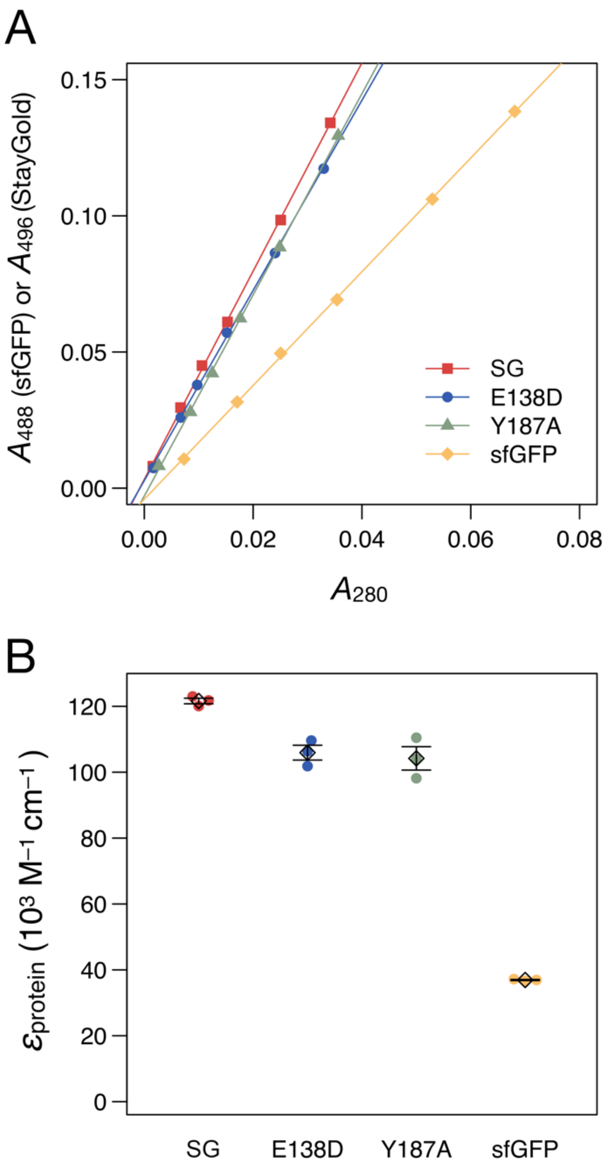

Figure S5: Extinction coefficient measurements for StayGold, mStayGold and sfGFP using the A280 nm method. (a) Representative plots of the chromophore absorption maxima versus absorbance at 280 nm for StayGold, mStayGold E138D, mStayGold Y187A and superfolder GFP (sfGFP). (b) Molar extinction coefficients derived for three independent measurements displayed with mean and standard error.

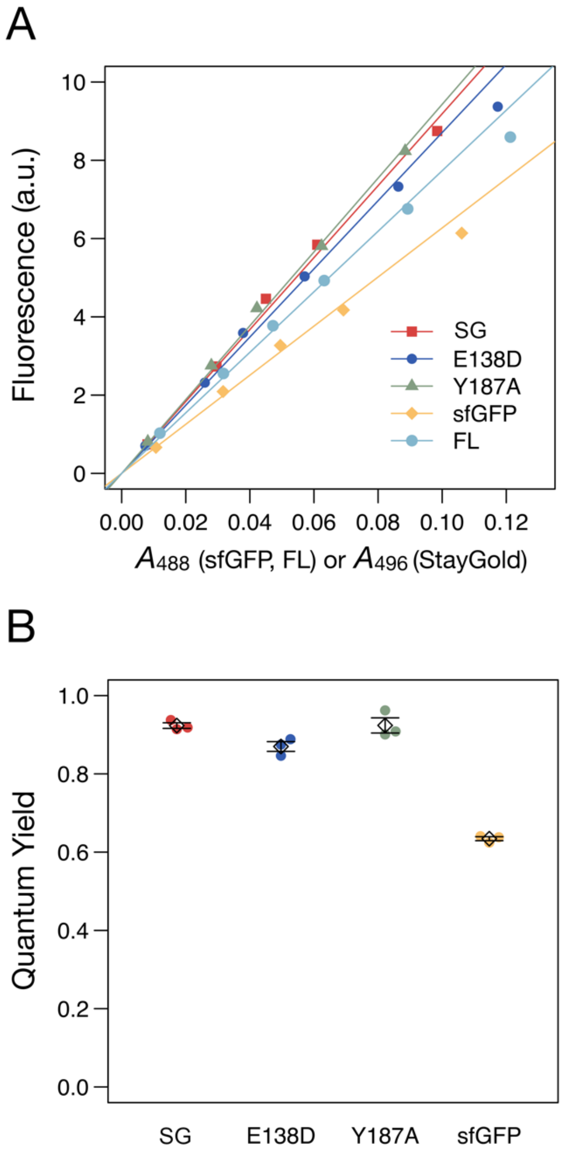

Figure S6: Quantum yield measurements for StayGold, mStayGold and sfGFP. (A) Representative plot of integrated fluorescence vs absorbance for StayGold, mStayGold (E138D), mStayGold (Y187A), superfolder GFP (sfGFP) and Fluorescein (FL). The gradient of each plot was used to derive quantum yields relative to that reported for fluorescein (Quantum yield 0.79). (B) Quantum yield values obtained for three independent measurements, each displayed with their mean and standard error.

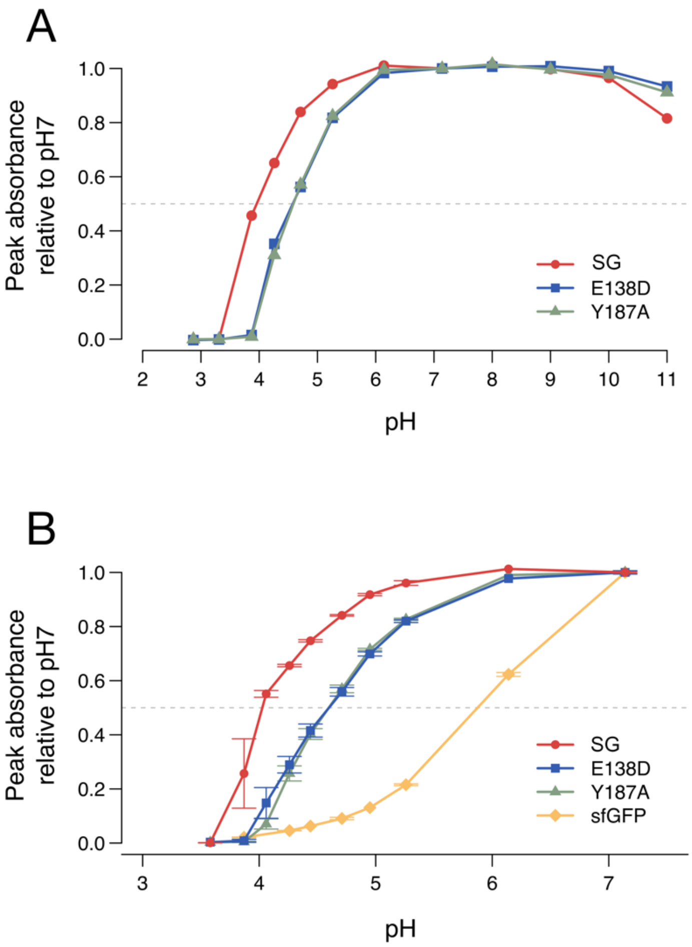

Figure S7: pH measurements and pK_a determinations. (a) Absorbance of StayGold and its derivatives measured at 496 nm under different pH conditions, relative to the value at neutral pH. (B) Additional mesurements at low pH for the purpose of pK_a determination. Absorbance at 496 nm (StayGold derivatives) or 488 nm (sfGFP) were measured under in a citrate/phosphate buffer system and normalised to the value at neutral pH. Results from three independent measurements are displayed with their mean and standard error. Apparent pK_a values are: StayGold 4.0; mStayGold E138D 4.6; mStayGold Y187A 4.0; and sfGFP 5.8.

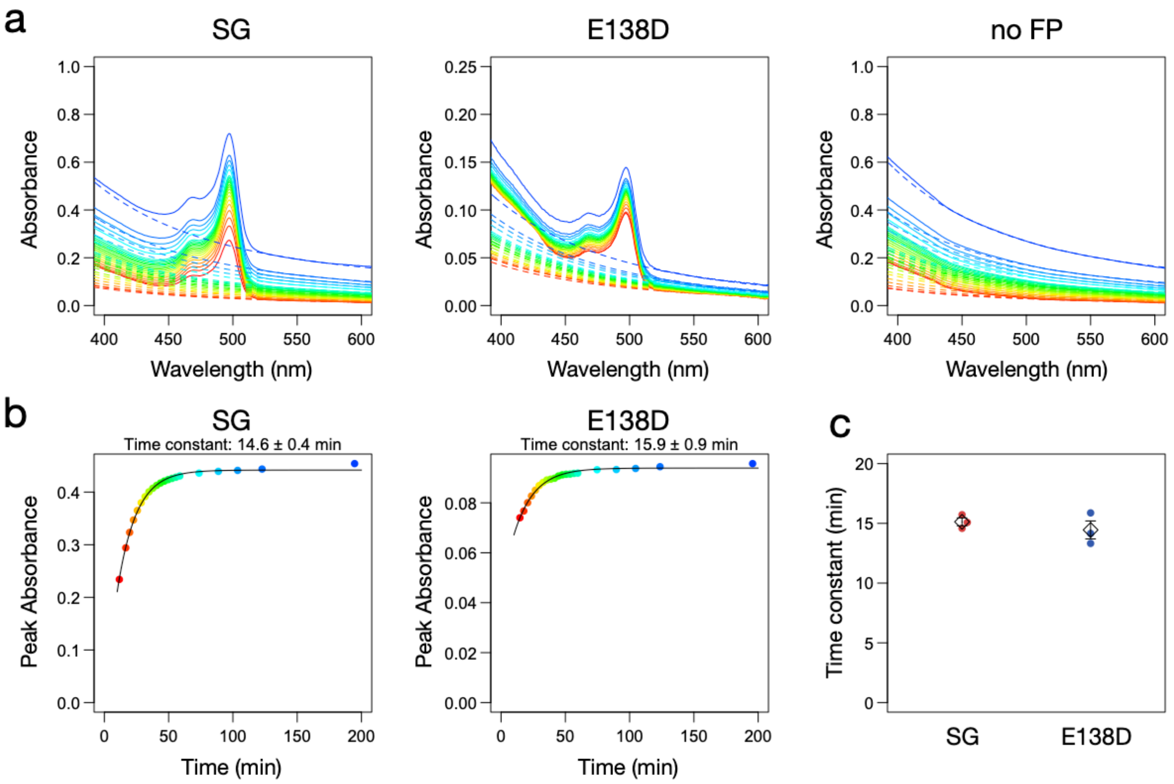

Figure S8: Oxidative maturation of the chromophore of StayGold in *E. coli* lysate.
(a) The absorbance spectra of the cleared cell lysate containing immature StayGold (SG), mStayGold (E138D), or a non-fluorescent protein (no FP) expressed under an anaerobic condition (solid lines). The colours (the same as in b) indicate the time in an aerobic condition, which started at cell lysis. The contribution of scattering (dashed lines), which gradually increased during incubation at room temperature independently of the fluorescent proteins (no FP), was estimated by extrapolating the absorbance between 525 nm and 575 nm. (b) The time course of chromophore maturation assessed by the peak absorbance corrected for the baseline scattering (closed circles). The rate of chromophore maturation, k, was estimated by fitting with a function of time, t, $f(t) = \beta_0 - \beta_1 \exp(-kt)$, in which β_0, β_1, and k are the parameters (solid line). (c) The experiment was repeated three times. The estimated values of the time constant (the inverse of the rate) were plotted (closed circles) with the mean (diamond) and the standard error (error bars) (15.1 ± 0.3 min and 14.5 ± 0.8 min for StayGold and mStayGold, respectively. There is no significant difference between the maturation times of StayGold and mStayGold (p-value of Welch's t-test is 0.4744).

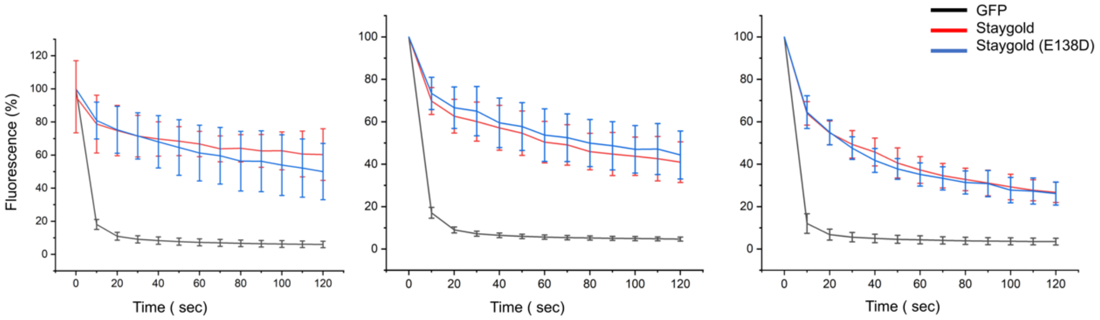

Figure S9: Additional repeat experiments for photostability measurements in live yeast. The three experiments shown are replicates of the experiment presented in **Fig. 2d** of the main manuscript. Each point represents the mean and standard deviation of the fluorescence intensity normalised to the first time point (n=20).

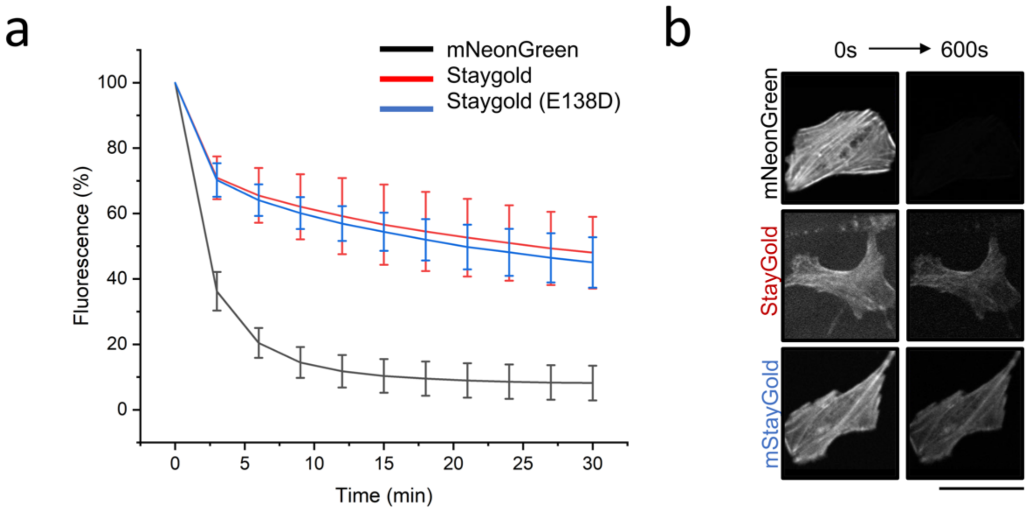

Figure S10: Photostability of mStayGold, StayGold, and measurements for fixed human RPE-1 cells. Each fluorescent protein was expressed as a fusion with human tropomyosin2 and the time-lapse imaging was performed after fixation. (a) Photostability comparison for StayGold, mStayGold, and mNeonGreen during imaging of fixed RPE-1 cells. The mean and standard deviation of the fluorescence intensity normalized with the first time point (n=30 cells for each construct collected over 3 independent experiments). (b) Representative images showing tropomyosin fluorescent protein fusions in fixed human cells for each fluorescent protein fusion (Scale bar 10µm).

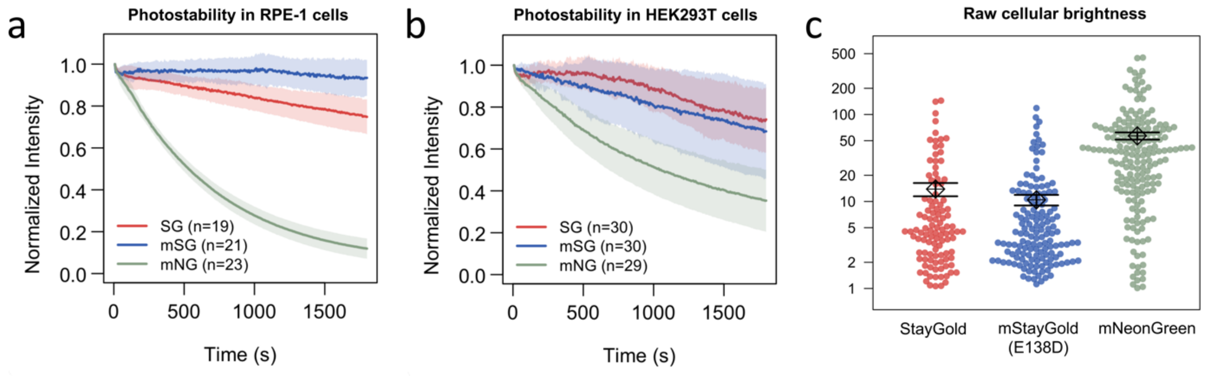

Figure S11: Photostability measurements for freely expressed StayGold, mStayGold and mNeonGreen in RPE-1 and HEK293T cells. (a). Photostability measurements in RPE-1 cells. (b) Photostability measurements in HEK293T cells. Solid lines represent the mean normalised intensity of cells expressing the indicated fluorescent protein and shaded areas represent the standard deviation. The number of the cells observed over three independent experiments is shown inset. (c) A comparison of cellular brightness for freely expressed StayGold, mStayGold, and mNeonGreen (n=107, 144, and 184, respectively, observed in one experiment). Data is plotted on a log scale. Each point represents the fluorescence intensity for a single RPE-1 cell. Mean intensity values are 14, 10 and 57 for StayGold, mStayGold (E138D) and mNeonGreen, respectively.

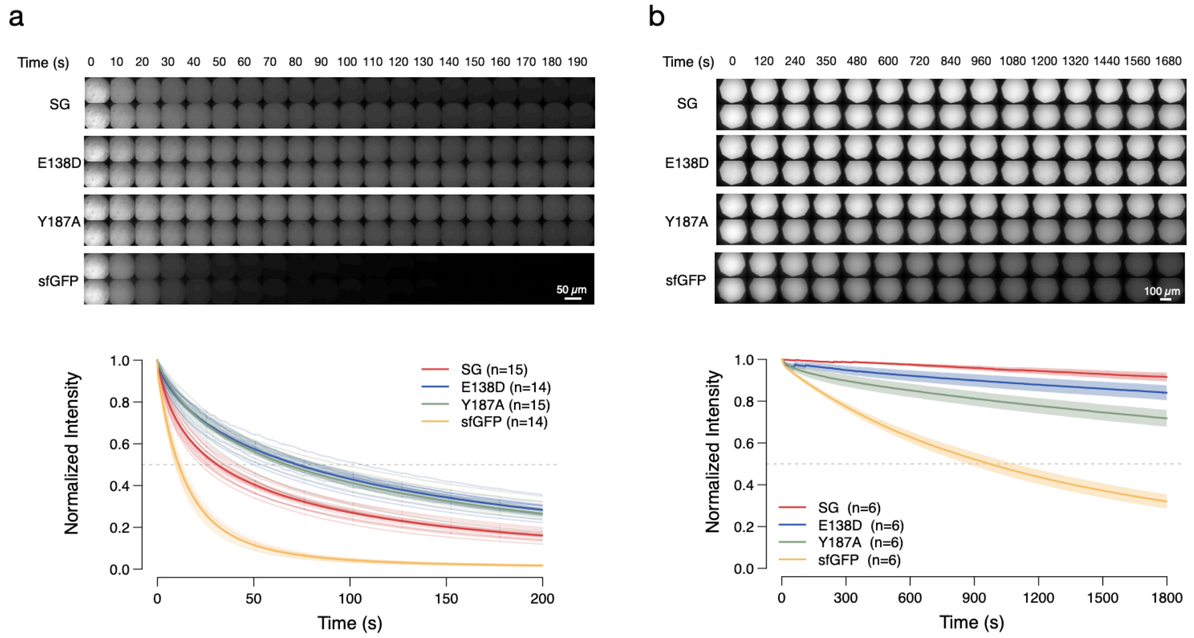

Figure S12: *In vitro* photostability measurements using purified fluorescent proteins immobilised in acrylamide gel. (a) Photobleaching of immobilised fluorescent proteins under 488 nm laser illumination. (b) Photobleaching of gel immobilised proteins illuminated by 480-500 nm light from a metal halide lamp. Background-subtracted fluorescence is normalised to the starting value. The mean of independent measurements was plotted (thick line) with either raw data (multiple thin lines) or the standard error (coloured area). The measured times-to-half-bleaching under laser illumination were: StayGold 31.6±1.5 seconds; mStayGold (E138D) 73.5±3.8 seconds; mStayGold Y187A 69.8±3.9; and sfGFP 10.9±0.3 seconds.

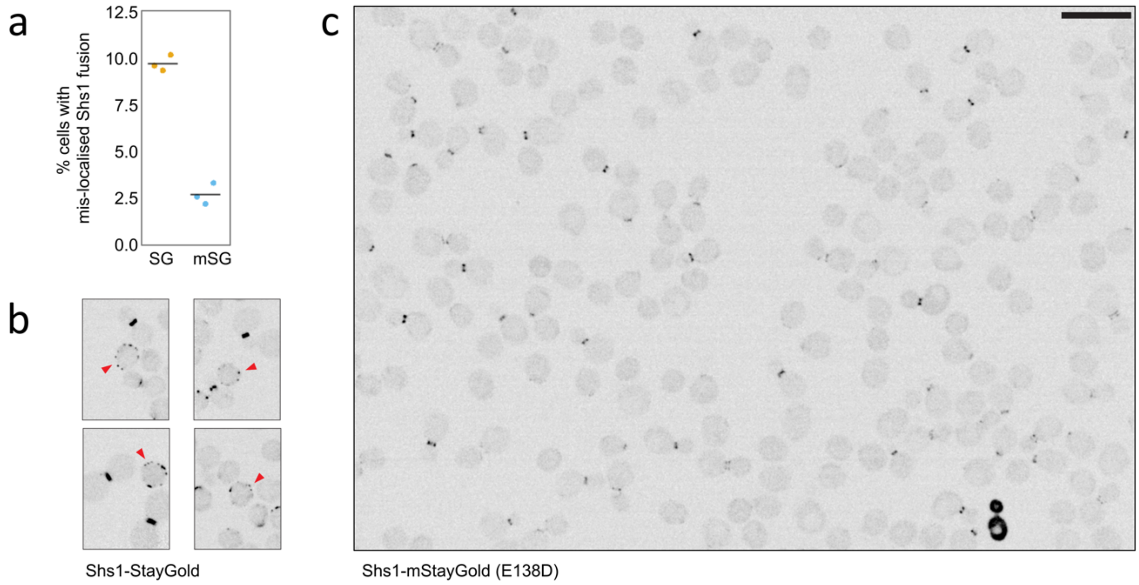

Figure S13: Mis-localisation of the budding yeast septin collar protein Shs1 using a StayGold fusion is corrected by introduction of the E138D monomerising mutation. (a) Percentage of cells with mis-localised Shs1-StayGold fusions for the original StayGold (SG) or the mStayGold carrying the E138D substitution (mSG). (b) Examples of aggregates formed using the original StayGold as a fusion tag for Septin Shs1. (c) Field of cells expressing the Shs1-mStayGold (E138D) variant showing correct labelling of the septal collar with very few aggregates. The three datapoints for the Shs1-StayGold fusion are based on counts of *n*=826, *n*=836 and *n*=1116 cells. The three datapoints for the Shs1-mStayGold(E138D) are underpinned by counts of *n*=329, *n*=460, and *n*=557 cells. Scale bar 10 µm.

a

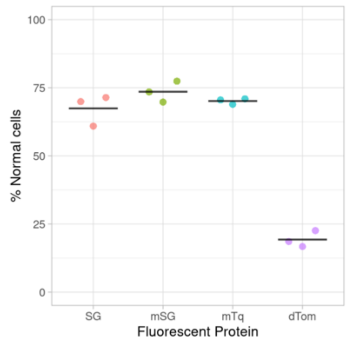

b

SG	mSG	mTq	dTom
70% (n=143)	70% (n=152)	69% (n=206)	19% (n=226)
71% (n=133)	77% (n=177)	71% (n=234)	23% (n=155)
61% (n=128)	73% (n=166)	71% (n=258)	17% (n=221)

c

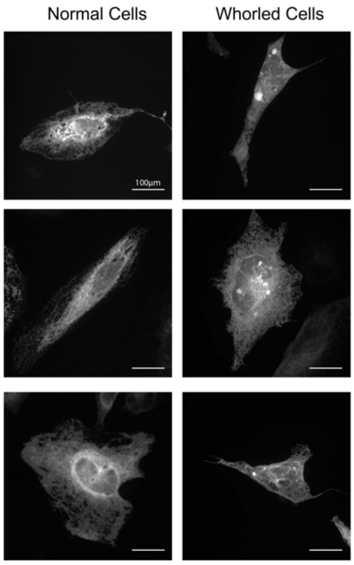

Figure S14: OSER assay data demonstrating *in vivo* monomerization of mStayGold. The indicated fluorescent protein was expressed as a cytoplasmic-side fusion to the endoplasmic reticulum signal anchor membrane protein (CytERM) and expressed in HeLa cells[5]. Fluorescent protein oligomerisation of such fusions induces abnormal structures of the endoplasmic reticulum ('whorls') that can be identified by microscopy (examples of 'normal' and 'whorled' cells are shown to the right of the figure. The percentage of normal cells is reported as an indicator of *in vivo* oligomerisation[5]. To provide context to the scores reported, we use dTomato and mNeonGreen as controls for dimerization and monomerization respectively. (a) Plotted OSER scores for three repeat experiments using StayGold (SG), mStayGold E138D (mSG), mTurquoise (mTq), and dTomato (dTom). (b) Tabulated OSER scores including the total number of cells analysed. (c) Examples of 'normal' and 'whorled' cells.

Supplemental References

1. Murshudov, G. N. *et al. REFMAC* 5 for the refinement of macromolecular crystal structures. *Acta Crystallogr. D Biol. Crystallogr.* **67**, 355–367 (2011).
2. Lovell, S. C. *et al.* Structure validation by Cα geometry: ϕ,ψ and Cβ deviation. *Proteins Struct. Funct. Bioinforma.* **50**, 437–450 (2003).
3. Hirano, M. *et al.* A highly photostable and bright green fluorescent protein. *Nat. Biotechnol.* **40**, 1132–1142 (2022).
4. Zacharias, D. A., Violin, J. D., Newton, A. C. & Tsien, R. Y. Partitioning of Lipid-Modified Monomeric GFPs into Membrane Microdomains of Live Cells. *Science* **296**, 913–916 (2002).
5. Costantini, L. M., Fossati, M., Francolini, M. & Snapp, E. L. Assessing the Tendency of Fluorescent Proteins to Oligomerize Under Physiologic Conditions: Fluorescent Protein Oligomerization Assay. *Traffic* **13**, 643–649 (2012).
