# Supplementary material for: A monomeric StayGold fluorescent protein
Source: Nat Biotechnol. 2023 Dec 11;42(9):1368–71. doi: 10.1038/s41587-023-02018-w (PMC11392804; doi:10.1038/s41587-023-02018-w)
Supplement: Supplementary file 1 [file 41587_2023_2018_MOESM2_ESM.pdf]

nature portfolio

Corresponding author(s): Mohan K. Balasubramanian, Allister Crow, Masanori Mishima

Last updated by author(s): Sep 26, 2023

Reporting Summary

Nature Portfolio wishes to improve the reproducibility of the work that we publish. This form provides structure for consistency and transparency in reporting. For further information on Nature Portfolio policies, see our Editorial Policies and the Editorial Policy Checklist.

Statistics

For all statistical analyses, confirm that the following items are present in the figure legend, table legend, main text, or Methods section.

n/a	Confirmed	
☐	☒	The exact sample size (*n*) for each experimental group/condition, given as a discrete number and unit of measurement
☐	☒	A statement on whether measurements were taken from distinct samples or whether the same sample was measured repeatedly
☐	☒	The statistical test(s) used AND whether they are one- or two-sided<br>*Only common tests should be described solely by name; describe more complex techniques in the Methods section.*
☒	☐	A description of all covariates tested
☐	☒	A description of any assumptions or corrections, such as tests of normality and adjustment for multiple comparisons
☐	☒	A full description of the statistical parameters including central tendency (e.g. means) or other basic estimates (e.g. regression coefficient) AND variation (e.g. standard deviation) or associated estimates of uncertainty (e.g. confidence intervals)
☐	☒	For null hypothesis testing, the test statistic (e.g. *F*, *t*, *r*) with confidence intervals, effect sizes, degrees of freedom and *P* value noted<br>*Give P values as exact values whenever suitable.*
☒	☐	For Bayesian analysis, information on the choice of priors and Markov chain Monte Carlo settings
☒	☐	For hierarchical and complex designs, identification of the appropriate level for tests and full reporting of outcomes
☒	☐	Estimates of effect sizes (e.g. Cohen's *d*, Pearson's *r*), indicating how they were calculated

Our web collection on statistics for biologists contains articles on many of the points above.

Software and code

Policy information about availability of computer code

Data collection	The GDA suite (for x-ray data collection - maintained by Diamond syncrotron).<br>Andor iQ3,<br>Micro-Manager 1.4.22,<br>Olympus xcellence rt 2.0,<br>Cary WinFLR,<br>Cary WinUV
Data analysis	Pymol 2.5.4,<br>CCP4 8.0.006,<br>Refmac 5.8.0352,<br>Aimless 0.7.8,<br>Phaser 2.8.3,<br>Parrot - 1.0.7,<br>Buccaneer - 1.6.12,<br>Rampage - 1.00.0,<br>Microsoft Excel 2023,<br>R version 4.3.1 (2023-06-16),<br>OriginPro 2023a,<br>Fiji 2.9.0/ImageJ 1.53t

For manuscripts utilizing custom algorithms or software that are central to the research but not yet described in published literature, software must be made available to editors and reviewers. We strongly encourage code deposition in a community repository (e.g. GitHub). See the Nature Portfolio guidelines for submitting code & software for further information.

Data

Policy information about availability of data

All manuscripts must include a data availability statement. This statement should provide the following information, where applicable:

- Accession codes, unique identifiers, or web links for publicly available datasets
- A description of any restrictions on data availability
- For clinical datasets or third party data, please ensure that the statement adheres to our policy

The structure of the StayGold dimer has been deposited at the protein databank (8BXT). Source data for biochemical and spectrometric experiments (Figures 2a, S2, S3, S4, S5, S6, S7, and S8) and numerical data from the microscopy-based photokinetic or photobiological studies (Figures 2d, S9, S10, S11, S12, S13, and S14) are available for download as a zip file. Due to the large size of the dataset, raw images from the microscopy-based studies (Figures 2d, S9, S10, S11, S12, S13, and S14) will be shared on request.

Research involving human participants, their data, or biological material

Policy information about studies with human participants or human data. See also policy information about sex, gender (identity/presentation), and sexual orientation and race, ethnicity and racism.

Reporting on sex and gender	N/A
Reporting on race, ethnicity, or other socially relevant groupings	N/A
Population characteristics	N/A
Recruitment	N/A
Ethics oversight	N/A

Note that full information on the approval of the study protocol must also be provided in the manuscript.

Field-specific reporting

Please select the one below that is the best fit for your research. If you are not sure, read the appropriate sections before making your selection.

☒ Life sciences ☐ Behavioural & social sciences ☐ Ecological, evolutionary & environmental sciences

For a reference copy of the document with all sections, see nature.com/documents/nr-reporting-summary-flat.pdf

Life sciences study design

All studies must disclose on these points even when the disclosure is negative.

Sample size	No explicit sample size calculation was performed. We followed community norms. For time-lapse imaging of photobleaching (Figures 2d, S9, S10, S11, S12), the time required for photobleaching was a major factor that limits the sample size for the practically achievable experimental design. In biochemical and spectrometric experiments (Figures S2, S3, S4, S5, S6, S7, and S8) each experiment was repeated at least three times. Size exclusion chromatography was performed in duplicate.
Data exclusions	No data were excluded.
Replication	The photobleaching microscopy in Figures 2d+S9 were independently repeated 4 times. For photobleaching in Figures S10 and S11, the biochemical and spectrometric experiments (Figures S2, S3, S4, S5, S6, S7b, and S8) and visual scoring (Figures S13 and S14), three independent experiments were performed and the consolidated data were presented. Measurement of the sensitivity to pH>7 (Figure S7a) was performed once. The in vitro photobleaching with laser (Figure S12a) was repeated three times and one of the three consistent results was shown. For bleaching with the halogen lamp (Figure S12b), one set of measurements (n=6 for each sample) was performed.
Randomization	For microscopy of the cells (Figures 2d, S9, S10, S11, S13 and S14), the imaged fields were randomly selected.
Blinding	Blinding was not used.

Reporting for specific materials, systems and methods

We require information from authors about some types of materials, experimental systems and methods used in many studies. Here, indicate whether each material, system or method listed is relevant to your study. If you are not sure if a list item applies to your research, read the appropriate section before selecting a response.

Materials & experimental systems

n/a	Involved in the study
☒	☐ Antibodies
☐	☒ Eukaryotic cell lines
☒	☐ Palaeontology and archaeology
☒	☐ Animals and other organisms
☒	☐ Clinical data
☒	☐ Dual use research of concern
☒	☐ Plants

Methods

n/a	Involved in the study
☒	☐ ChIP-seq
☒	☐ Flow cytometry
☒	☐ MRI-based neuroimaging

Eukaryotic cell lines

Policy information about cell lines and Sex and Gender in Research

Cell line source(s)	hTERT RPE-1 cells (CRL-4000), HeLa (CCL-2) and HEK293T (CRL-3216) were all sourced from ATCC.
Authentication	None
Mycoplasma contamination	Cells used in this study were tested for mycoplasma with negative results
Commonly misidentified lines (See ICLAC register)	HEK293T
