# Supplementary material for: A monomeric StayGold fluorescent protein
Source: Nat Biotechnol. 2023 Dec 11;42(9):1368–71. doi: 10.1038/s41587-023-02018-w (PMC11392804; doi:10.1038/s41587-023-02018-w)
Supplement: Supplementary file 2 [file 41587_2023_2018_MOESM4_ESM.zip › FigureS4/output/ECchro_beeswarm.pdf]

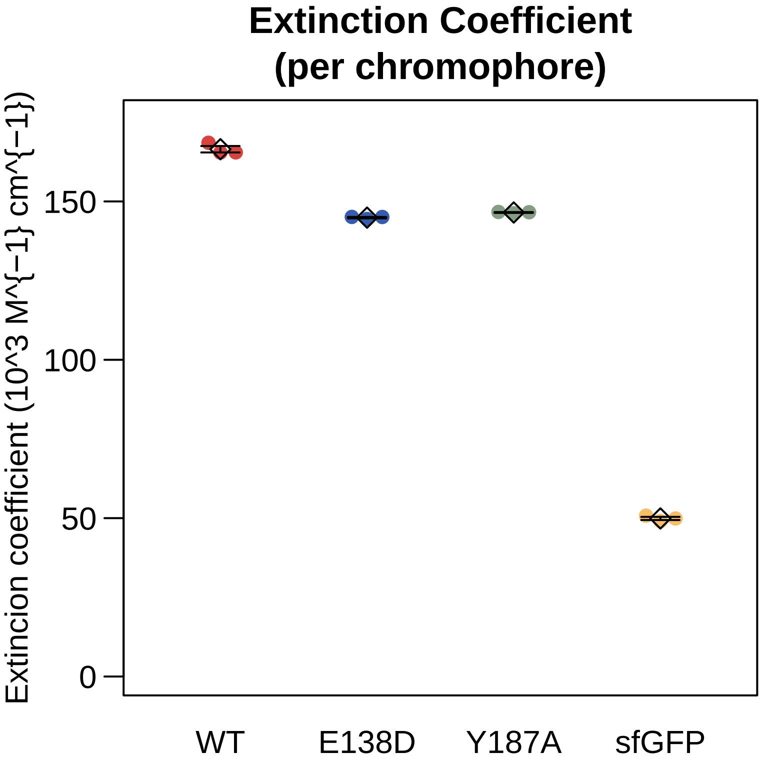

Extinction Coefficient
(per chromophore)
Extincion coefficient (10^3 M^{−1} cm^{−1})
150
100
50
0
WT
E138D
Y187A
sfGFP
