# Supplementary material for: A monomeric StayGold fluorescent protein
Source: Nat Biotechnol. 2023 Dec 11;42(9):1368–71. doi: 10.1038/s41587-023-02018-w (PMC11392804; doi:10.1038/s41587-023-02018-w)
Supplement: Supplementary file 2 [file 41587_2023_2018_MOESM4_ESM.zip › FigureS7/ABS_wider_pH.pdf]

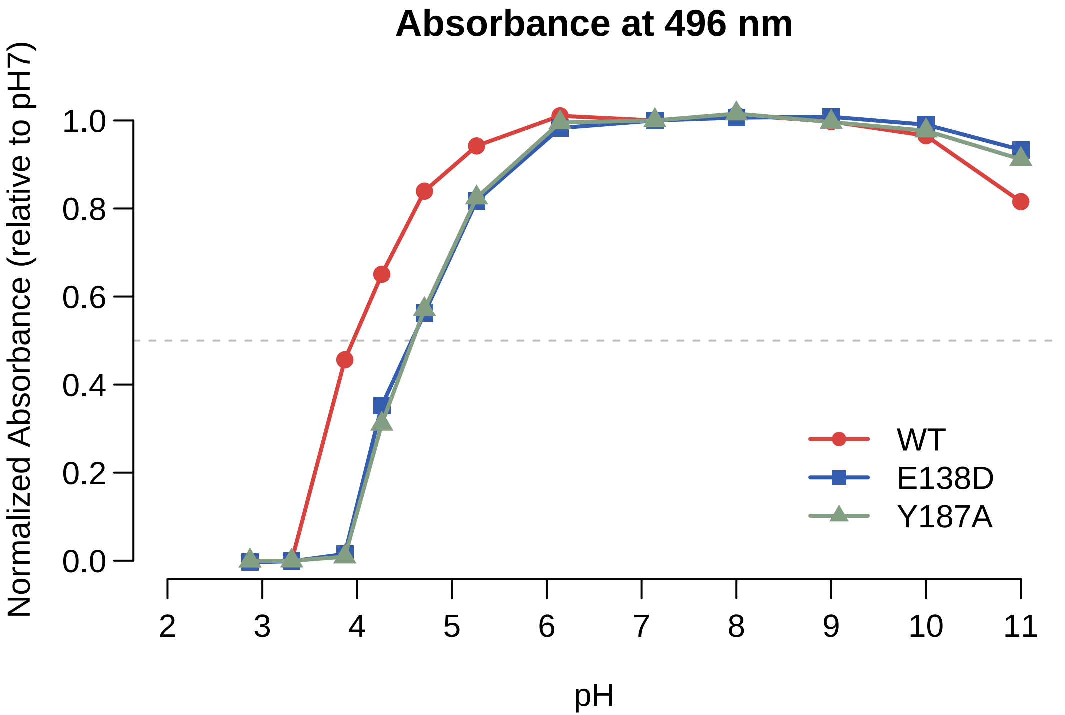

Absorbance at 496 nm
Normalized Absorbance (relative to pH7)
1.0
0.8
0.6
0.4
0.2
0.0
2
3
4
5
6
7
8
9
10
11
pH
WT
E138D
Y187A
