# Supplementary material for: A monomeric StayGold fluorescent protein
Source: Nat Biotechnol. 2023 Dec 11;42(9):1368–71. doi: 10.1038/s41587-023-02018-w (PMC11392804; doi:10.1038/s41587-023-02018-w)
Supplement: Supplementary file 2 [file 41587_2023_2018_MOESM4_ESM.zip › FigureS12/FigureS12b/bleaching.pdf]

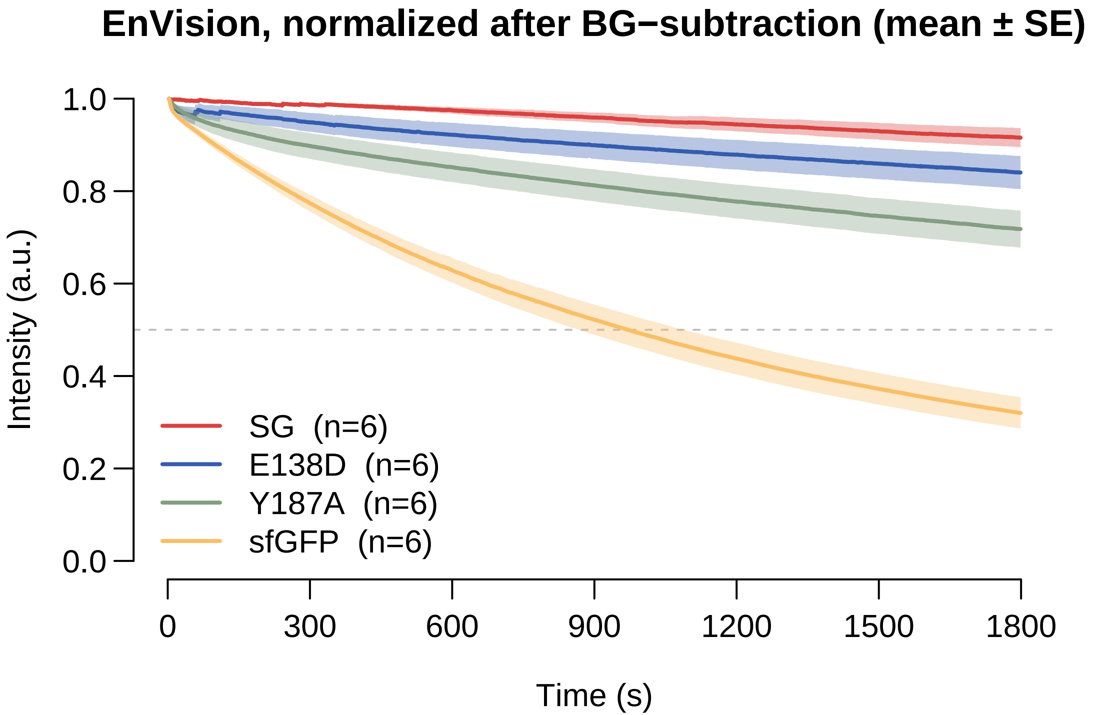
EnVision, normalized after BG−subtraction (mean ± SE)
1.0
0.8
0.6
0.4
0.2
0.0
Intensity (a.u.)
SG (n=6)
E138D (n=6)
Y187A (n=6)
sfGFP (n=6)
0
300
600
900
1200
1500
1800
Time (s)
