# Supplementary material for: A monomeric StayGold fluorescent protein
Source: Nat Biotechnol. 2023 Dec 11;42(9):1368–71. doi: 10.1038/s41587-023-02018-w (PMC11392804; doi:10.1038/s41587-023-02018-w)
Supplement: Supplementary file 2 [file 41587_2023_2018_MOESM4_ESM.zip › FigureS12/FigureS12a/bleaching.pdf]

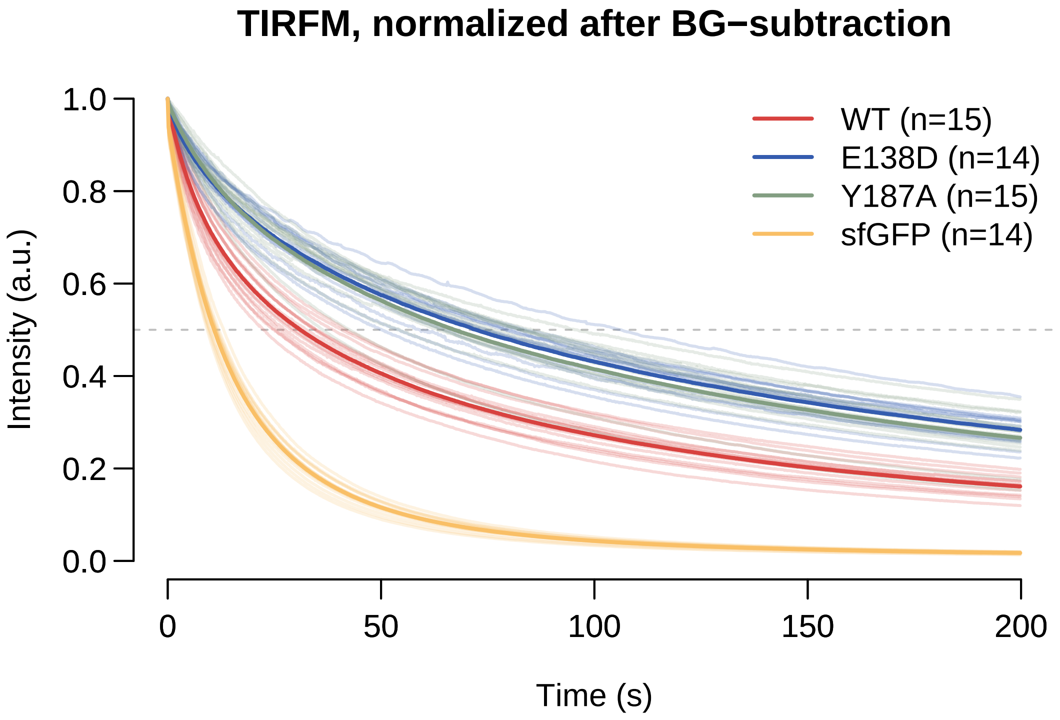

TIRFM, normalized after BG−subtraction
WT (n=15)
E138D (n=14)
Y187A (n=15)
sfGFP (n=14)
Intensity (a.u.)
1.0
0.8
0.6
0.4
0.2
0.0
0
50
100
150
200
Time (s)
