# Supplementary material for: A monomeric StayGold fluorescent protein
Source: Nat Biotechnol. 2023 Dec 11;42(9):1368–71. doi: 10.1038/s41587-023-02018-w (PMC11392804; doi:10.1038/s41587-023-02018-w)
Supplement: Supplementary file 2 [file 41587_2023_2018_MOESM4_ESM.zip › FiguresS5+S6/output/QY_beeswarm.pdf]

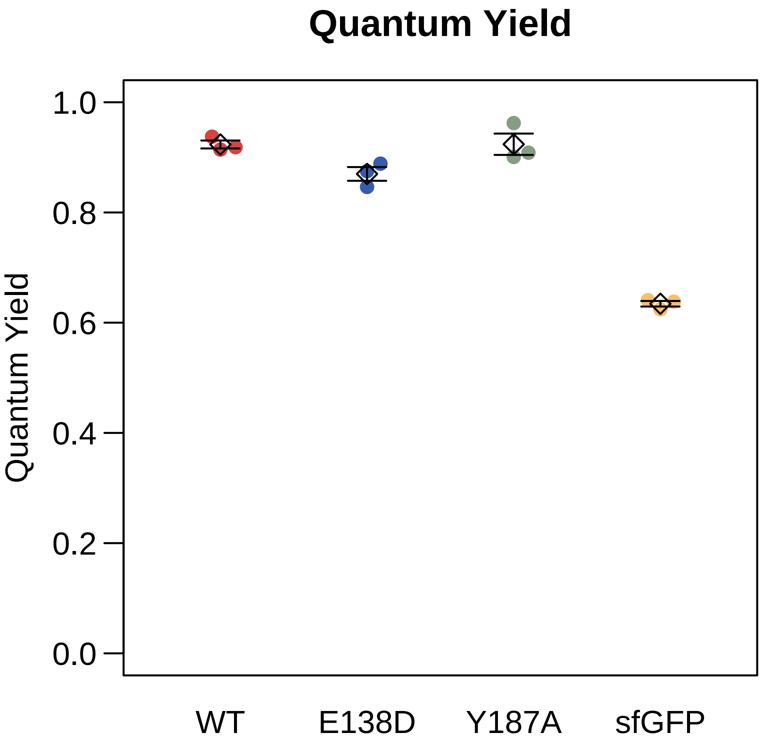
Quantum Yield
Quantum Yield
1.0
0.8
0.6
0.4
0.2
0.0
WT
E138D
Y187A
sfGFP
