# Supplementary material for: A monomeric StayGold fluorescent protein
Source: Nat Biotechnol. 2023 Dec 11;42(9):1368–71. doi: 10.1038/s41587-023-02018-w (PMC11392804; doi:10.1038/s41587-023-02018-w)
Supplement: Supplementary file 2 [file 41587_2023_2018_MOESM4_ESM.zip › FiguresS5+S6/output/A280-peak_exp1.pdf]

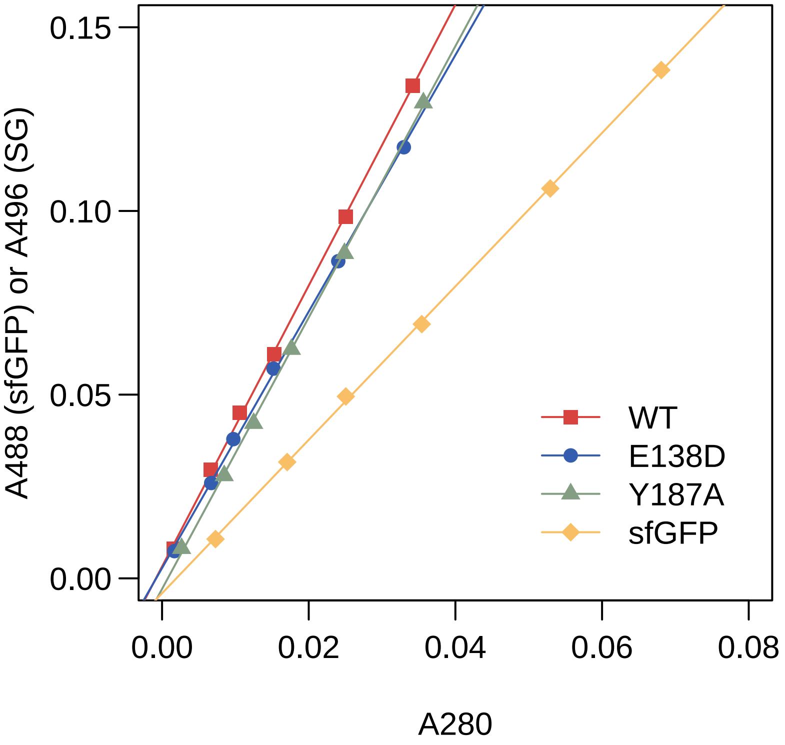

0.15
0.10
0.05
0.00
A488 (sfGFP) or A496 (SG)
0.00
0.02
0.04
0.06
0.08
A280
WT
E138D
Y187A
sfGFP
